# Melatonin-Mediated Intracellular Insulin during 2-Deoxy-d-glucose Treatment Is Reduced through Autophagy and EDC3 Protein in Insulinoma INS-1E Cells

**DOI:** 10.1155/2016/2594703

**Published:** 2016-07-17

**Authors:** Han Sung Kim, Tae-Young Han, Yeong-Min Yoo

**Affiliations:** ^1^Department of Biomedical Engineering, Yonsei-Fraunhofer Medical Device Laboratory, Yonsei University, Wonju, Gangwon-do 26493, Republic of Korea; ^2^Fraunhofer Institute IKTS-MD, Maria-Reiche-Straße 2, 01109 Dresden, Germany

## Abstract

2-DG triggers glucose deprivation without altering other nutrients or metabolic pathways and then activates autophagy via activation of AMPK and endoplasmic reticulum (ER) stress. We investigated whether 2-DG reduced intracellular insulin increased by melatonin via autophagy/EDC3 in insulinoma INS-1E cells. p-AMPK and GRP78/BiP level were significantly increased by 2-DG in the presence/absence of melatonin, but IRE1*α* level was reduced in 2-DG treatment. Levels of p85*α*, p110, p-Akt (Ser473, Thr308), and p-mTOR (Ser2481) were also significantly reduced by 2-DG in the presence/absence of melatonin. Mn-SOD increased with 2-DG plus melatonin compared to groups treated with/without melatonin alone. Bcl-2 was decreased and Bax increased with 2-DG plus melatonin. LC3II level increased with 2-DG treatment in the presence/absence of melatonin. Intracellular insulin production increased in melatonin plus 2-DG but reduced in treatment with 2-DG with/without melatonin. EDC3 was increased by 2-DG in the presence/absence of melatonin. Rapamycin, an mTOR inhibitor, increased GRP78/BiP and EDC3 levels in a dose-dependent manner and subsequently resulted in a decrease in intracellular production of insulin. These results suggest that melatonin-mediated insulin synthesis during 2-DG treatment involves autophagy and EDC3 protein in rat insulinoma INS-1E cells and subsequently results in a decrease in intracellular production of insulin.

## 1. Introduction

Pancreatic *β*-cells are the only source of the peptide hormone insulin, which is essential for stimulating glucose uptake in peripheral tissue and inhibiting glucose production in liver. Pancreatic *β*-cell dysfunction and death result from a combination of genetic predisposition and exposure to environmental risk factors and contribute to type 2 diabetes (T2D) [1]. The endoplasmic reticulum (ER) contains the protein-folding machinery for secretory proteins and is therefore crucial for insulin biosynthesis. Disruption of *β*-cell ER function by elevated free fatty acid levels and insulin resistance can result in an imbalance in protein homeostasis and ER stress, which has been recognized as an important mechanism for T2D [2, 3]. Recent reports have shown that autophagy is activated in response to ER stress in pancreatic *β*-cells [3–5].

AMP-activated protein kinase (AMPK) is a central metabolic regulator which balances energy production through the AMP : ATP ratio in eukaryotic cells and functions as a therapeutic target for anticancer and metabolic diseases in diabetes mellitus and lipid metabolism [6–8]. AMPK appears to be involved in insulin signaling pathways by positively regulating Akt/Protein Kinase B (PKB) in endothelial cells and cardiomyocytes [9, 10]. However, AMPK activation has also been reported to negatively affect Akt/PKB and insulin production [11]. Activation or phosphorylation of AMPK is associated with activation or phosphorylation of Akt/PKB and mammalian target of rapamycin (mTOR) for insulin synthesis or secretion in pancreatic *β*-cells [12, 13].

Melatonin can control insulin secretion both in vivo and in vitro via MT1 or MT2 receptors [14, 15], Gi protein [16], cyclic AMP-response element-binding protein (CREB) [17], and Gq proteins/phospholipase C/IP3 [18]. Circadian rhythms are influenced by melatonin and also induce a phase shift in insulin secretion [19]. Melatonin directly influences diabetes and associated metabolic diseases [20]. The genes and proteins involved in direct regulation of insulin biosynthesis and secretion in pancreatic *β*-cells have been reported: IRE1*α* [21], Sirt1 [22], and Sirt4 [23]. Recently, melatonin was shown to directly influence insulin biosynthesis and secretion under ER stress via the RNA-binding protein human antigen D (HuD) in rat pancreatic INS-1E cells, suggesting that nuclear HuD may be an important negative regulator in insulin production and secretion [13]. However, an interaction between the enhancer of mRNA decapping complex 3 (EDC3) [24] and insulin production has not been reported.

The glucose analogue 2-deoxy-d-glucose (2-DG) is metabolized by hexokinase and acts as an inhibitor of glycolysis [25]. 2-DG triggers glucose deprivation without altering other nutrients or metabolic pathways [26] and then activates autophagy via activation of AMPK [27] and endoplasmic reticulum (ER) stress [28]. Therefore, 2-DG appears to be an ideal tool to understand the interactions between autophagy and ER stress. In this study, we provide the first demonstration that 2-DG reduces intracellular insulin which was increased by melatonin via autophagy and EDC3 in insulinoma INS-1E cells.

## 2. Materials and Methods

### 2.1. Cell Culture

INS-1E cells, a clonal pancreatic *β*-cell line, were obtained from Professor Claes B. Wollheim and were cultured in RPMI 1640 medium (Invitrogen, Carlsbad, CA, USA) containing 11 mM glucose supplemented with 10 mM HEPES (pH 7.3), 10% heat-inactivated fetal bovine serum (FBS; Invitrogen), 50 *μ*M *β*-mercaptoethanol, 1 mM sodium pyruvate, 50 *μ*g/mL penicillin, and 100 *μ*g/mL streptomycin at 37°C with 5% CO_2_ in a humidified incubator.

### 2.2. Treatment with 2-DG, Melatonin, and Rapamycin

INS-1E cells were cultured in RPMI 1640 medium plus 2% heat-inactivated FBS in a 37°C and 5% CO_2_ incubator, with or without melatonin (Sigma-Aldrich, St. Louis, MO, USA) and/or 2-DG (5 mM) (Sigma-Aldrich) for 24 hr, or with rapamycin (20 to 80 nM) (Calbiochem, San Diego, MO, USA) for 24 hr.

### 2.3. Western Blot Analysis

Cells were harvested, washed twice with ice-cold phosphate buffered saline (PBS), and then resuspended in 20 mM Tris-HCl buffer (pH 7.4) containing protease inhibitors (0.1 mM phenylmethylsulfonyl fluoride, 5 *μ*g/mL aprotinin, 5 *μ*g/mL pepstatin A, and 1 *μ*g/mL chymostatin) and phosphatase inhibitors (5 mM Na_3_VO_4_ and 5 mM NaF). Whole cell lysate was prepared using 20 strokes of a Dounce homogenizer, followed by centrifugation at 13,000 ×g for 20 min at 4°C. The protein concentration was determined using the BCA assay (Sigma). Proteins (40 *μ*g) were separated by 12% sodium dodecyl sulfate-polyacrylamide gel electrophoresis (SDS-PAGE) and the detection of insulin proteins was performed by 16.5% tricine SDS-PAGE. These gels transferred onto a polyvinylidene difluoride (PVDF) membrane. The membrane was incubated with antibodies (diluted as indicated in brackets) directed against the following proteins: p-AMPK and AMPK (1 : 500) (Santa Cruz Biotechnology); IRE1*α* (1 : 500) (Santa Cruz Biotechnology); p-PERK (1 : 1000) (Cell Signaling Technology, Beverly, MA, USA); GRP78/BiP (1 : 1000) (Cell Signaling Technology); p85*α* and p85*β* (1 : 500) (Santa Cruz Biotechnology); p110 (1 : 500) (Santa Cruz Biotechnology); p-Akt (T308, S473) and Akt (1 : 1000) (Cell Signaling Technology); p-mTOR (Ser2448, 2481) and mTOR (1 : 1000) (Cell Signaling Technology); Cu/Zn-SOD (1 : 500) (Santa Cruz Biotechnology); Mn-SOD (1 : 500) (Santa Cruz Biotechnology); catalase (1 : 500) (Santa Cruz Biotechnology); Bcl-2 (1 : 500) (Santa Cruz Biotechnology); Bax (1 : 500) (Santa Cruz Biotechnology); insulin (1 : 500) (Santa Cruz Biotechnology); EDC3 (1 : 500) (Santa Cruz Biotechnology); and Actin (1 : 1000) (Assay Designs, Ann Arbor, MI, USA). Immunoreactive proteins were visualized by exposure to X-ray film. Protein bands were analyzed by image-scanning, and optical density was measured using ImageJ analysis software (version 1.37, Wayne Rasband, NIH, Bethesda, MD, USA). The data were corrected for background subtraction and normalized by including Actin as an internal control.

### 2.4. Statistical Analysis

Significant differences were detected by ANOVA, followed by Tukey's test for multiple comparisons. Analysis was performed using the Prism Graph Pad v4.0 (Graph Pad Software Inc., San Diego, CA, USA). Values are expressed as means ± SD of at least three separated experiments, in which case a representative experiment is depicted in the figures. *p* values < 0.05 were considered statistically significant.

## 3. Results

### 3.1. Expressions of p-AMPK, GRP78/BiP, IRE1*α*, and p-PERK Proteins

**Figure 1:**
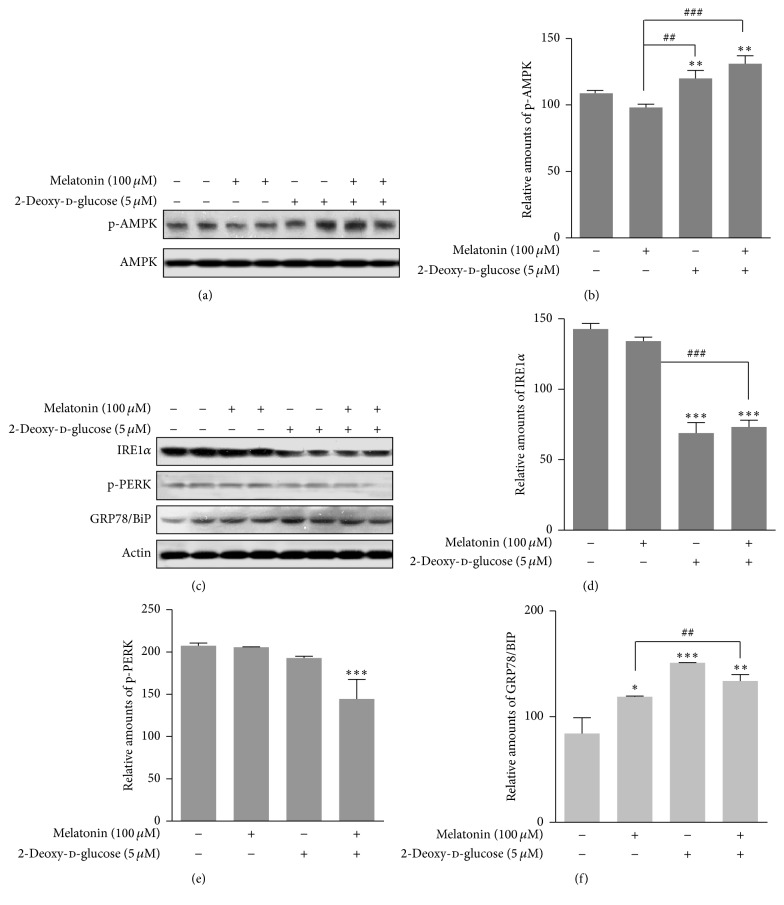
Phosphorylation of AMPK, IRE1*α* protein expression, phosphorylation of PERK, and GRP78/BiP protein expression in the presence or absence of melatonin and/or 2-DG in rat insulinoma INS-1E cells. INS-1E cells were incubated in RPMI 1640 medium supplemented with 2% FBS with/without melatonin (100 *μ*M) and/or 2-DG (5 mM) for 24 hr at 37°C with 5% CO_2_. p-AMPK expression was analyzed by Western blot (a, c). The relative amount of p-AMPK (b), IRE1*α* protein (d), p-PERK (e), and GRP78/BiP protein (f) was quantified as described in Section 2. Data represent mean ± SD of three experiments (*n* = 3). ^*∗*^ *p* < 0.05, ^*∗∗*^ *p* < 0.01, and ^*∗∗∗*^ *p* < 0.001 versus 2% FBS; ^##^ *p* < 0.01, ^###^ *p* < 0.001, melatonin versus melatonin and/or 2-DG.

We investigated whether 2-DG reduced insulin synthesis which had been increased by melatonin via autophagy in rat insulinoma INS-1E cells. 2-DG activates autophagy via reactive oxygen species-mediated activation of AMPK [27] and ER stress [28]. Therefore, we first investigated whether 2-DG and/or melatonin induced phosphorylation of AMPK and ER stress in rat insulinoma INS-1E cells. Phosphorylation of AMPK was significantly increased by treatment with 2-DG in the presence or absence of melatonin, compared to treatment with melatonin or FBS only (Figures 1(a) and 1(b)). ER stress marker GRP78/BiP protein was also significantly increased by treatment with 2-DG in the presence or absence of melatonin (Figures 1(c) and 1(d)), but expression of IRE1*α* protein was reduced (Figures 1(c) and 1(d)) and p-PERK protein expression was not significantly affected by 2-DG treatment (Figures 1(c) and 1(e)).

### 3.2. Expressions of p-PI3K, Akt/PKB, and mTOR Proteins

**Figure 2 fig2:**
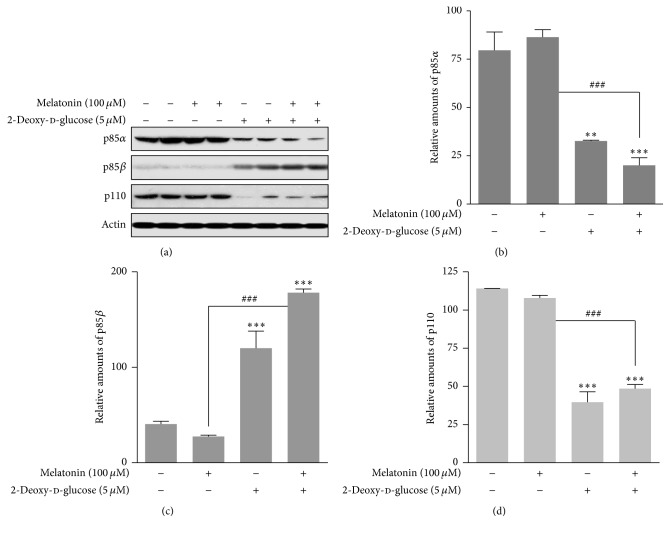
Expression of p85*α*, p85*β*, and p110 proteins in the presence or absence of melatonin and/or 2-DG in rat insulinoma INS-1E cells. INS-1E cells were incubated in RPMI 1640 medium supplemented with 2% FBS with/without melatonin (100 *μ*M) and/or 2-DG (5 mM) for 24 hr at 37°C with 5% CO_2_. p85*α*, p85*β*, and p110 proteins were analyzed by Western blot (a). The relative amounts of p85*α* (b), p85*β* (c), and p110 proteins (d) were quantified in Section 2. Data represent mean ± SD of three experiments (*n* = 3). ^*∗∗*^ *p* < 0.05 and ^*∗∗∗*^ *p* < 0.001 versus 2% FBS; ^###^ *p* < 0.001, melatonin versus melatonin and/or 2-DG.

**Figure 3 fig3:**
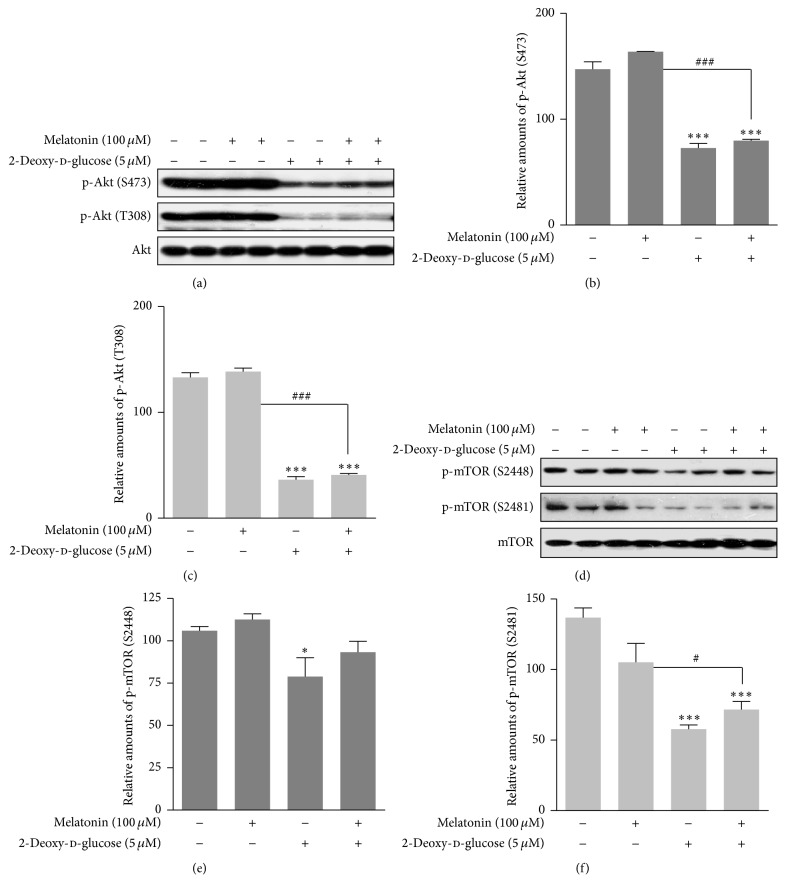
Phosphorylation of Akt (Ser473), Akt (Thr308), mTOR (Ser2448), and mTOR (Ser2481) proteins in the presence or absence of melatonin and/or 2-DG in rat insulinoma INS-1E cells. INS-1E cells were incubated in RPMI 1640 medium supplemented with 2% FBS with/without melatonin (100 *μ*M) and/or 2-DG (5 mM) for 24 hr at 37°C with 5% CO_2_. p-Akt was analyzed by Western blot (a, d). The relative amounts of p-Akt (Ser473) (b), p-Akt (Thr308) (c), mTOR (Ser2448) (e), and mTOR (Ser2481) (f) were quantified in Section 2. Data represent mean ± SD of three experiments (*n* = 3). ^*∗*^ *p* < 0.05, ^*∗∗∗*^ *p* < 0.01 versus 2% FBS; ^#^ *p* < 0.05, ^###^ *p* < 0.001, melatonin versus melatonin and/or 2-DG.

We determined how 2-DG and/or melatonin affected known regulators of insulin signaling, phosphoinositide 3-kinase (PI3K), Akt/PKB, and mTOR, in rat insulinoma INS-1E cells. Class IA PI3K is composed of a heterodimer of p110 catalytic subunit and p85 regulatory subunit [29]. 2-DG treatment with or without melatonin caused a significant reduction in expression of PI3K subunit p85*α* and p110 and a significant increase in p85*β* protein expression (Figures 2(a)–2(d)). 2-DG also significantly decreased expression of p-Akt (Ser473, Thr308) (Figures 3(a)–3(c)) and p-mTOR (Ser2481) (Figures 3(d)–3(f)) proteins in the presence or absence of melatonin. These results indicated that ER stress in the presence of 2-DG or 2-DG-plus melatonin negatively regulates the PI3K/Akt/mTOR pathway.

### 3.3. Expressions of SOD, Catalase, Bcl-2, and Bax Proteins

**Figure 4 fig4:**
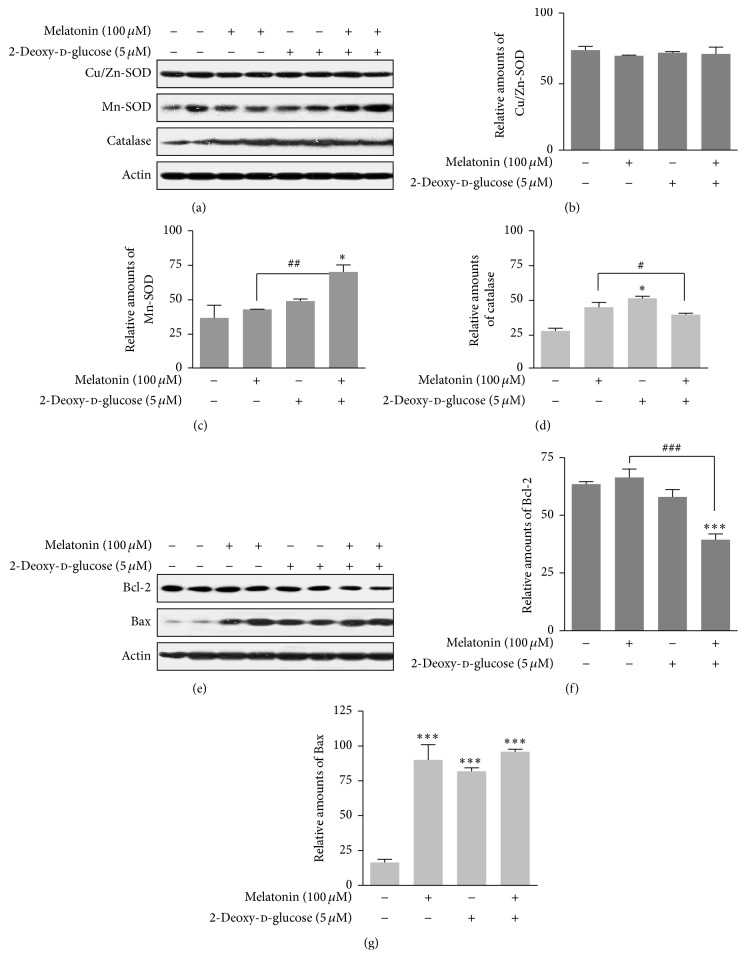
Expression of Cu/Zn-SOD, Mn-SOD, catalase, Bcl-2, and Bax proteins in the presence or absence of melatonin and/or 2-DG in rat insulinoma INS-1E cells. INS-1E cells were incubated in RPMI 1640 medium supplemented with 2% FBS with/without melatonin (100 *μ*M) and/or 2-DG (5 mM) for 24 hr at 37°C with 5% CO_2_. Cu/Zn-SOD, Mn-SOD, and catalase proteins were analyzed by Western blot (a, e). The relative amounts of Cu/Zn-SOD (b), Mn-SOD (c), catalase proteins (d), Bcl-2 (f), and Bax proteins (g) were quantified in Section 2. Data represent mean ± SD of three experiments (*n* = 3). ^*∗*^ *p* < 0.05, ^*∗∗∗*^ *p* < 0.001 versus 2% FBS; ^#^ *p* < 0.05, ^##^ *p* < 0.01, and ^###^ *p* < 0.001, melatonin versus melatonin and/or 2-DG.

Next, to characterize intracellular and mitochondrial conditions according to antioxidant and prooxidant proteins, we examined how 2-DG and/or melatonin affected superoxide dismutase (SOD), catalase, Bcl-2, and Bax proteins in rat insulinoma INS-1E cells. Cu/Zn-SOD was not significantly affected (Figures 4(a) and 4(b)), but Mn-SOD protein expression was increased by treatment with 2-DG plus melatonin compared to groups treated with/without melatonin alone or the nontreated control group (Figures 4(a) and 4(c)). Catalase protein expression was decreased by 2-DG treatment in the presence of melatonin (Figures 4(a) and 4(d)). Bcl-2 protein expression was decreased by treatment with 2-DG plus melatonin compared to treatment with/without melatonin (Figures 4(e) and 4(f)), and Bax protein expression was increased in cells treated with 2-DG and/or melatonin, compared to nontreated control group (Figures 4(e) and 4(g)). These results suggested that 2-DG or 2-DG-plus melatonin influenced mitochondria, thus resulting in a change of antioxidant and prooxidant protein levels.

### 3.4. Expressions of LC3II, Insulin, and EDC3 Proteins

**Figure 5 fig5:**
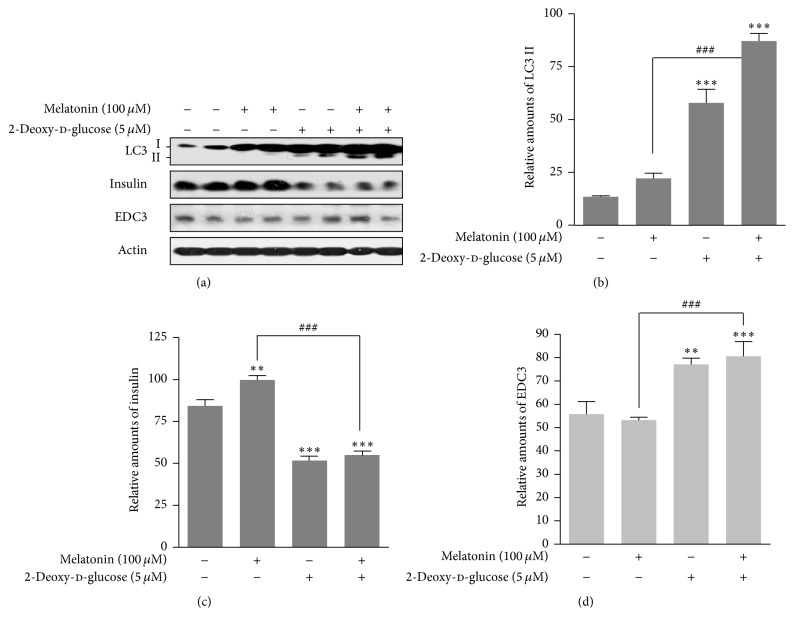
Expression of LC3, insulin, and EDC3 proteins in the presence or absence of melatonin and/or 2-DG in rat insulinoma INS-1E cells. INS-1E cells were incubated in RPMI 1640 medium supplemented with 2% FBS with/without melatonin (100 *μ*M) and/or 2-DG (5 mM) for 24 hr at 37°C with 5% CO_2_. LC3, Insulin, and EDC3 proteins were analyzed by Western blot (a). The relative amounts of LC3 (b), insulin (c), and EDC3 proteins (d) were quantified in Section 2. Data represent mean ± SD of three experiments (*n* = 3). ^*∗∗*^ *p* < 0.01, ^*∗∗∗*^ *p* < 0.001 versus 2% FBS; ^###^ *p* < 0.001, melatonin versus melatonin and/or 2-DG.

Subsequently, we investigated whether 2-DG reduced insulin synthesis which had been increased by melatonin via EDC3 in rat insulinoma INS-1E cells. Expression of autophagy markers LC3 II was increased by 2-DG treatment in the presence or absence of melatonin (Figures 5(a) and 5(b)). Insulin synthesis was increased by melatonin treatment compared to nontreated melatonin group, but it was reduced by treatment with 2-DG (Figures 5(a) and 5(c)). Expression of EDC3, a negative regulator in insulin synthesis, was increased by 2-DG in the presence or absence of melatonin (Figures 5(a) and 5(d)), suggesting that the decrease in intracellular insulin synthesis was associated with an increase of autophagy and EDC3 protein expression.

### 3.5. The Relationship of Insulin Production with Autophagy and EDC3 Protein

**Figure 6:**
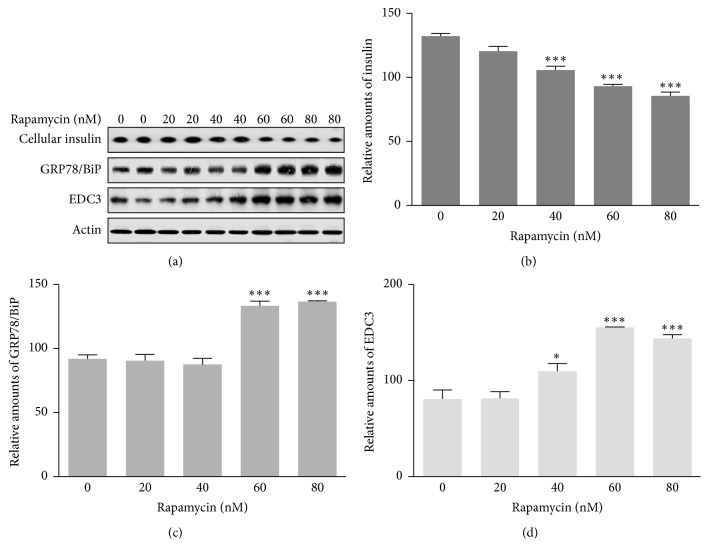
Expression of insulin, GRP78/BiP, and EDC3 proteins with or without rapamycin treatment in rat insulinoma INS-1E cells. INS-1E cells were incubated in RPMI 1640 medium supplemented with 2% FBS with/without rapamycin (20 to 80 nM) for 24 hr at 37°C with 5% CO_2_. Insulin, GRP78/BiP, and EDC3 proteins were analyzed by Western blot (a). The relative amounts of insulin (b), GRP78/BiP (c), and EDC3 proteins (d) were quantified in Section 2. Data represent mean ± SD of three experiments (*n* = 3). ^*∗*^ *p* < 0.05, ^*∗∗∗*^ *p* < 0.001 versus 1% FBS.

Lastly, to confirm the relationship of insulin production with autophagy and EDC3 protein, we investigated whether the mTOR inhibitor, rapamycin, induced autophagy and expression of GRP78/BiP and EDC3 proteins in rat insulinoma INS-1E cells. Rapamycin treatment resulted in an increase in GRP78/BiP (Figures 6(a) and 6(c)) and EDC3 proteins in a dose-dependent manner (Figures 6(a) and 6(d)) and a subsequent decrease in insulin production (Figures 6(a) and 6(b)). These results suggested that melatonin-mediated insulin synthesis during 2-DG treatment involved autophagy-induced ER stress and EDC3 protein in rat insulinoma INS-1E cells, subsequently resulting in a decrease in insulin protein biosynthesis.

## 4. Discussion

ER dysfunction has been implicated in insulin resistance and is an important cause of T2D [2–4]. Autophagy in ER stress-induced pancreatic *β* cells plays a role as an important regulator of insulin production and secretion [2–5, 13]. In addition, autophagy in pancreatic *β* cells is essential to maintain normal morphology, cell mass, and function of *β*-cells and is regarded as a crucial stress response factor to protect *β*-cells under insulin-resistant states [4, 5]. The glucose analog 2-DG acts as a glycolytic inhibitor, leading to ER stress and an unfolded protein response [26–28]. Although 2-DG-induced ER stress has been shown to activate autophagy, the precise mechanism is not fully understood. Here we demonstrated the relevance of ER stress-mediated autophagy caused by 2-DG for insulin synthesis in pancreatic *β* cells. Our previous report showed that ER stress caused by melatonin, especially in the presence of thapsigargin, decreased intracellular insulin biosynthesis and that extracellular secretion of insulin may be regulated by melatonin in rat insulinoma INS-1E cells [13]. Here we found that melatonin reduced insulin production in the presence of 2-DG via autophagy-induced ER stress in rat insulinoma INS-1E cells (Figures 1(c)–1(f) [Fig fig2] [Fig fig3] [Fig fig4] [Fig fig5] and 6(a)–6(d)). mTOR inhibitor, rapamycin, induced autophagy and then subsequently decrease in insulin production (Figure 6). Therefore, autophagy can regulate insulin production at the cellular level through physiological and biochemical changes of ER homeostasis involving the unfolded protein response [30, 31].

GRP78/BiP is localized in the ER and its expression is increased by environmental stresses in many types of cells. Other studies have showed that conditions including glucose deprivation, 2-DG treatment, and hypoxia or increase of intracellular Ca^2+^ concentration induce GRP78/BiP expression [32–35]. Our previous research demonstrated that expression of the ER-stress protein GRP78/BiP is significantly decreased by tunicamycin/melatonin treatment and remains almost unperturbed with thapsigargin/melatonin treatment when compared to control/melatonin in rat pancreatic INS-1E cells [13]. This suggests that GRP78/BiP protein can be regulated by several cellular stresses which perturb ER function and homeostasis, including tunicamycin, thapsigargin, and calcium ionophore A23187 [36]. 2-DG is an especially potent inducer of GRP78/BiP protein expression [37]. In this study we found that melatonin significantly regulated insulin production through the expression of GRP78/BiP protein which induced ER stress under conditions of 2-DG or 2-DG plus melatonin treatment in rat insulinoma INS-1E cells (Figures 1(c) and 1(f)). Therefore, GRP78/BiP accompanied with ER stress is a crucial element in insulin biosynthesis and secretion in rat pancreatic *β*-cells [38].

The interaction between 2-DG and the AMPK/mTOR signal pathway has not been reported in pancreatic *β* cells. Activation of AMPK and inactivation of mTOR signal pathway have only been studied under conditions of nutrient deprivation, including glucose and/or leucine starvation, in pancreatic beta-cells, rat skeletal muscle cells, and hepatocytes [12, 13, 39, 40]. Gleason et al. [12] reported that because glucose and amino acids stimulate insulin release from pancreatic *β* cells, AMPK represents an important factor for control of *β*-cell function. In addition, the activation/phosphorylation of AMPK in *β*-cells by low glucose correlates with inactivation of the mTOR pathway as a cellular sensor for nutritional conditions in *β* cells [12, 41]. The other condition, that is, lack of amino acid leucine, causes an increase in the activation/phosphorylation of AMPK and a decrease in the activation of mTOR pathway [12, 39, 40]. It is therefore unlikely that the ability of AMPK and the mTOR signal pathway to sense both glucose and amino acids plays a role in regulation of insulin synthesis or secretion [12, 41]. 2-DG induces glucose deprivation and results in the activation/phosphorylation of AMPK [26, 27]. In our study, phosphorylation of AMPK was significantly increased (Figures 1(a) and 1(b)) and phosphorylation of mTOR (Ser2481) was significantly decreased (Figures 3(d) and 3(f)) by 2-DG in the presence or absence of melatonin, showing that *β*-cells do indeed possess a mechanism by which glucose derivation can suppress the mTOR signal pathway via activation of AMPK. This result suggests that a connection between AMPK and mTOR in the *β*-cell is of particular relevance to insulin synthesis/secretion during 2-DG treatment. The finding that AMPK and the mTOR signal pathway can sense both glucose and amino acids may apply to 2-DG treatment conditions in the regulation of insulin synthesis or its secretion from *β*-cells.

The genes/proteins involved in insulin biosynthesis and secretion in pancreatic *β*-cells have been studied: IRE1*α* [21], Sirt1 [22], Sirt4 [23], and HuD [13]. The positive regulators of insulin biosynthesis and secretion are IRE1*α*, Sirt1, and WSF1, whereas Eny2, Sirt4, and HuD are negative regulators. Our recent study suggests that nuclear HuD under ER stress conditions with thapsigargin and melatonin functions as an important negative regulator of insulin production from rat pancreatic INS-1E cells [13]. However, our current study demonstrated that HuD under autophagy and ER stress with 2-DG and melatonin could not negatively regulate insulin production (data not shown). Instead, EDC3 was involved in insulin synthesis during 2-DG/melatonin or rapamycin treatment and subsequently induced a decrease in insulin protein (Figures 5 and 6). An interaction between EDC3 and insulin production has not yet reported [24]. Our study is the first to suggest that EDC3 functions as a negative regulator of insulin production in rat pancreatic INS-1E cells.

Glucose deprivation by mechanisms including 2-DG induces the production of reactive oxygen species (ROS) to activate AMPK in pancreatic *β* cells [42]. Hypoxia and hyperoxia trigger AMPK activation via mitochondrial ROS production [43]. These studies support a role for AMPK as an important redox sensor through a mitochondrial ROS mechanism. We showed that 2-DG and/or melatonin affected SOD, catalase, Bcl-2, and Bax proteins in rat insulinoma INS-1E cells (Figure 4), suggesting that 2-DG or 2-DG plus melatonin influences antioxidant and prooxidant proteins through a mechanism involving mitochondrial ROS.

Class IA PI3K is composed of a p110 catalytic subunit and a p85 regulatory subunit and plays a pivotal role in insulin signaling [29, 44]. We found that 2-DG treatment significantly reduced expression of both PI3K subunits in the presence or absence of melatonin (Figure 2). This result indicated that the p85 proteins may bind and stabilize the p110 subunit [44].

We report that melatonin may directly regulate the intracellular biosynthesis of insulin in the presence of 2-DG in rat insulinoma INS-1E cells. Intracellular insulin production may be partially regulated by the expression of ER stress GRP78/BiP-induced autophagy and EDC3 protein under conditions of 2-DG treatment.

## 5. Conclusions

These results suggest that melatonin-mediated insulin synthesis during 2-DG treatment involves autophagy and EDC3 protein in rat insulinoma INS-1E cells and subsequently results in a decrease in intracellular production of insulin.
